# Elegant Mouth Wash

**Published:** 1888-02

**Authors:** 


### Elegant Mouth-Wash.

Edina sends a sample of a mouth-wash, half a teaspoonful of which in a wineglassfull of water is used to refresh the mouth. It is a pale crimson and transparent solution, with the odor of oil of wintergreen. Its composition is fairly represented by the following formula:

Oil of wintergreen.......................................... ʒj
Oil of peppermint.......................................... ♏xv
Rose-aniline hydrochlorate (or magenta).......... gr. ss
Water.......................................... ℥ss
Glycerine,.......................................... ℥iij
Rectified spirit to.......................................... Oj

Dissolve the oils in the spirit, and the rose-aniline in the water; mix the latter solution with the glycerine, and pour it into the perfumed spirit. Mix.—*Chemist and Druggist.*

